# The effect of ensiled paulownia leaves in a high-forage diet on ruminal fermentation, methane production, fatty acid composition, and milk production performance of dairy cows

**DOI:** 10.1186/s40104-022-00745-9

**Published:** 2022-08-12

**Authors:** Haihao Huang, Dorota Lechniak, Malgorzata Szumacher-Strabel, Amlan Kumar Patra, Martyna Kozłowska, Pawel Kolodziejski, Min Gao, Sylwester Ślusarczyk, Daniel Petrič, Adam Cieslak

**Affiliations:** 1 Department of Animal Nutrition, Poznań University of Life Sciences, Wołyńska 33, 60-637 Poznań, Poland; 2 Department of Genetics and Animal Breeding, Poznań University of Life Sciences, Wolynska 33, Poznań, 60-637 Poland; 3 Department of Animal Nutrition, West Bengal University of Animal and Fishery Sciences, 37 K. B. Sarani, Kolkata, India; 4 Institute of Genetics and Animal Biotechnology, Polish Academy of Sciences, Postępu 36A, 05-552, Magdalenka, Warsaw, Poland; 5 Department of Animal Physiology, Biochemistry and Biostructure, Poznan University of Life Sciences, Wolynska 35, Poznan, 60-637 Poland; 6 Department of Pharmaceutical Biology and Botanic Garden of Medicinal Plants, Wrocław Medical University, Wrocław, 50-556 Poland; 7 Institute of Animal Physiology, Centre of Biosciences of Slovak Academy of Sciences, Šoltésovej 4-6, 040-01 Košice, Slovak Republic; 8 Department of Animal Nutrition, Faculty of Veterinary Medicine and Animal Science, Poznań University of Life Sciences, Poznań, 60-637 Poland

**Keywords:** Dairy cow, Fatty acid composition in milk, Methane emission, Paulownia leaves

## Abstract

**Background:**

The use of industrial by-products rich in bioactive compounds as animal feeds can reduce greenhouse gas production. Paulownia leaves silage (PLS) was supplemented to dairy cows' diet and evaluated in vitro (Exp. 1; Rusitec) and in vivo (Exp. 2, cannulated lactating dairy cows and Exp. 3, non-cannulated lactating dairy cows). The study investigated the PLS effect on ruminal fermentation, microbial populations, methane production and concentration, dry matter intake (DMI), and fatty acid (FA) proportions in ruminal fluid and milk.

**Results:**

Several variables of the ruminal fluid were changed in response to the inclusion of PLS. In Exp. 1, the pH increased linearly and quadratically, whereas ammonia and total volatile fatty acid (VFA) concentrations increased linearly and cubically. A linear, quadratic, and cubical decrease in methane concentration was observed with increasing dose of the PLS. Exp. 2 revealed an increase in ruminal pH and ammonia concentrations, but no changes in total VFA concentration. Inclusion of PLS increased ruminal propionate (at 3 h and 6 h after feeding), isovalerate, and valerate concentrations. Addition of PLS also affected several populations of the analyzed microorganisms. The abundances of protozoa and bacteria were increased, whereas the abundance of archaea were decreased by PLS. Methane production decreased by 11% and 14% in PLS-fed cows compared to the control in Exp. 2 and 3, respectively. Exp. 3 revealed a reduction in the milk protein and lactose yield in the PLS-fed cows, but no effect on DMI and energy corrected milk yield. Also, the PLS diet affected the ruminal biohydrogenation process with an increased proportions of C18:3 *cis*-9 *cis*-12 *cis*-15, conjugated linoleic acid, C18:1 *trans-*11 FA, polyunsaturated fatty acids (PUFA), and reduced n6/n3 ratio and saturated fatty acids (SFA) proportion in milk. The relative transcript abundances of the 5 of 6 analyzed genes regulating FA metabolism increased.

**Conclusions:**

The dietary PLS replacing the alfalfa silage at 60 g/kg diet can reduce the methane emission and improve milk quality with greater proportions of PUFA, including conjugated linoleic acid, and C18:1 *trans-*11 along with reduction of SFA.

**Graphical Abstract:**

Graphical abstract of the experimental roadmap

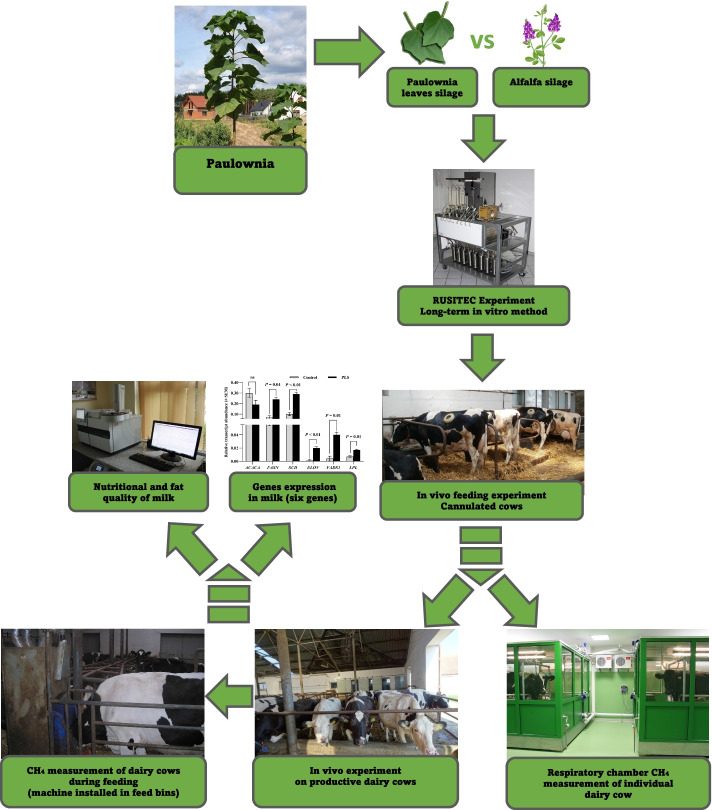

**Supplementary Information:**

The online version contains supplementary material available at 10.1186/s40104-022-00745-9.

## Highlights

• Paulownia leaves silage (PLS) fed to dairy cows decreased CH_4 _emission.

• Dietary PLS inhibited the methanogen community and increased selected bacteria populations.

• Diet containing PLS elevated propionate proportion and decreased acetate to propionate ratio in the rumen fluid.

• Transcript abundance of genes regulating fatty acid metabolism in the milk somatic cells were increased by PLS.

• Dietary PLS elevated proportion of unsaturated fatty acids, conjugated linoleic acid, and reduced n-6/n-3 ratio in the milk.

**Supplementary Information:**

The online version contains supplementary material available at 10.1186/s40104-022-00745-9.

## Introduction

Forage is one of the prerequisites to meet nutritional requirements of ruminants. Besides the forage like alfalfa silage, some by-products like pomegranate silage can be effectively utilized to protect the environment [1]. Trees of the *Paulownia* genus are known for their rapid growth [2] and thus an abundant source of wood [3]. A vast biomass of leaves becomes available as a by-product of wood processing. Leaves are rich in protein (average 175 g/kg DM), and hence can be used as an alternative source of forage for ruminants [4]. The nutritional value of Paulownia has been investigated in several studies [5–7]. Paulownia leaves are rich in bioactive compounds (BAC) such as phenolic acids and flavonoids as well as in fatty acids (FA) [4]. Besides, ensilaged paulownia leaves contain a higher concentration of BAC compared to its fresh material [4]. Our previous in vitro experiment showed that inclusion of either fresh or ensiled paulownia leaves lowered methane production by reducing the number of methanogens while improving the basic ruminal fermentation characteristics [4, 8]. Considering a high phenolic content of paulownia leaves, they can be a valuable component of dairy cow diet with an additional profit attributed to the improvement of the milk quality by altering its FA profile [4]. We hypothesized that paulownia leaves may beneficially alter ruminal fermentation variables such as pH or methane production, ammonia and volatile fatty acid concentration due to presence of phenolic compounds in PLS. Besides, the inclusion of PLS into the diet of dairy cows may affect milk composition by altering its FA profile by modifying ruminal biohydrogenation. The improvement of milk quality may result from an increase in n-3 PUFA concentration and thus a reduced n-6/n-3 ratio. However, the effect of dietary use of paulownia leaves silage (PLS) on milk production, methane mitigation, and ruminal fermentation has not been studied in dairy cows. To verify the hypothesis, we utilized the ensiled leaves of *Paulownia tomentosa* × *Paulownia fortunei* hybrid plants in an experimental design, which included in vitro and in vivo conditions. We aimed at investigating whether PLS affects (i) methane production and microbial population in vitro as well as (ii) ruminal fermentation parameters, methane production, and milk production and composition in vivo in dairy cows.

## Material and methods

### Diets and supplements

For the preparation of PLS, paulownia leaves (along with some twigs) were lopped from the trees (three-year-old plantations) at the end of May when the trees were shaped. Leaves were wilted for 8 h and were ensiled using the biological additive (Agricol Sil, Microferm, UK) in 60 kg plastic drums (diameter 40 cm and height 61 cm) according to Huang et al. [4]. The alfalfa was harvested at the second cut at the beginning of June. The alfalfa fresh materials after wilting approximately to 35% of dry matter were chopped to a particle length circa 2.5 cm and ensiled using the same biological additive as paulownia silage. One gram of the additive contained 10^11^ colony-forming units of *Lactobacillus plantarum* DSMZ 16,627 and *Pediococcus acidilactici* NCIMB 30,005, as well as an enzyme-producing strain, *Lactobacillus paracasei* NCIMB 3015. The closed drums were stored for 8-9 weeks in the case of both plants. The chemical composition of PLS and AS is presented in Table 1. The following fermentation parameters of PLS and AS were observed: pH, 4.75 vs. 4.50; NH_3_-N, 79.4 vs. 32.3 g/kg total nitrogen; lactic acid, 21.2 vs. 34.9 g/kg DM; acetic acid, 7.04 vs. 19.1 g/kg DM; propionic acid, 1.29 vs. 1.09 g/kg DM; butyric acid, 1.19 vs. 2.43 g/kg DM, respectively.

**Table 1:** Ingredients and chemical composition of experimental diets (*n* = 4) used in Rusitec system and in vivo experimets^a^ and chemical composition of paulownia leaves silage (PLS; *n* = 4) and alfalfa silage (AS; *n* = 4)

Item	PLS	AS	Treatments^b^			
			**CON**	**PLS, g/kg DM**		
				**20**	**40**	**60**
Ingredient composition, g/kg DM						
Corn silage	-	-	388	385	386	386
Alfalfa silage	-	-	82	68	47	26
Paulownia silage	-	-	0	21	39	60
Meadow grass silage	-	-	91	90	90	90
Beet pulp	-	-	103	103	103	103
Brewer’s grain	-	-	95	94	95	95
Concentrate^c^	-	-	119	118	119	119
Rapeseed meal	-	-	108	107	107	107
Mineral and vitamin premix^d^	-	-	14	14	14	14
Forage to concentrate ratio	-	-	76:24	76:24	76:24	76:24
Chemical composition^e^, g/kg DM						
DM, g/kg as fed	279	299	425	423	422	419
OM	869	874	909	907	904	900
aNDF	359	350	347	346	349	351
CP	174	218	161	160	158	158
EE	27.5	20.0	27.1	29.8	30.5	29.5
Total phenolic compounds^f^	60.0	-	0.00	1.20	2.40	3.60
VEM^g^	785	667	948	945	943	942
Fatty acid composition, g/100 g total FA						
C12:0	0.19	2.18	0.31	0.24	0.31	0.31
C14:0	0.71	1.90	0.47	0.40	0.50	0.48
C16:0	27.4	26.5	22.3	21.3	21.2	21.5
C16:1 *cis*-9	3.89	1.36	0.74	0.78	1.03	0.99
C18:0	4.16	4.01	2.63	2.55	2.16	2.82
C18:1 *cis*-9	5.26	4.76	20.3	19.7	18.9	17.8
C18:2 *cis*-9*.cis*-12	16.2	20.4	44.4	45.2	44.6	44.7
C18:3 *cis*-9.*cis*-12*.cis*-15	42.2	38.8	8.85	9.83	11.3	11.4

^a^In the in vitro experiments the diets were as a total mixed ration (TMR)

^b^*CON* control diet, *PLS* paulownia leaves silage diet, PLS was used at 20, 40 and 60 g/kg DM of diet replacing alfalfa silage

^c^Declared to contain (as g/kg of DM in concentrate) OM (910), aNDFom (240), CP (17.5), and EE (31)

^d^Declared to contain (g/kg of DM) Na (123), Ca (100), Mg (45), P (42), K (20), S (18), Co (14), Cu (5.0), Zn (2.8), Mn (1.4), Fe (1.05), F (0.42), I (0.028), Se (0.018), biotin (0.008); (IU/kg), vitamin A (200,000), vitamin D_3_ (40,000), and vitamin E (1200)

^e^*DM* dry matter, *OM* organic matter, *aNDF* neutral detergent fiber analyzed with α-amylase, *CP* crude protein, *EE*: ether extract. Additionally, in the case of PLS and AS, the following fermentation parameters were performed: pH, 4.75 vs. 4.50; NH_3_-N, 79.4 vs. 32.3 g/kg total nitrogen; lactic acid, 21.2 vs. 34.9 g/kg DM; acetic acid, 7.04 vs. 19.1 g/kg DM; propionic acid, 1.29 vs. 1.09 g/kg DM; butyric acid, 1.19 vs. 2.43 g/kg DM, respectively

^f^The content of total phenolic compounds have been calculated based on previous study of Huang et al. [4]

^g^VEM = feed unit net energy lactation; calculated using the FeedExpert software

### In vitro experiment (Exp. 1)

The in vitro experiment was conducted using the Rusitec system equipped with four fermenters of 1 L volume each, following the procedures described by Szczechowiak et al. [9]. Rumen fluid and solid digesta for the in vitro experiment were collected 3 h before the morning feeding from four rumen-cannulated (Bar Diamond, Parma, Idaho, USA) multiparous Polish Holstein–Friesian dairy cows (630 ± 25 kg body weight) at their 3^rd^ month of lactation. The following diets were tested: control diet (CON) and three PLS diets. The CON diet contained the following forages: corn silage (388 g/kg DM), alfalfa silage (82 g/kg DM), and meadow grass silage (91 g/kg DM; Table 1). The PLS diets contained paulownia silage that replaced alfalfa silage at 25%, 50%, and 75%, which corresponded to the PLS content in the diet at the level of 20, 40, and 60 g/kg DM (Table 1). Ruminal fluid donor cows received the CON diet twice a day ad libitum (Table 1). To meet the nutrient requirements of mid-lactation dairy cows (600 kg body weight, 110 d in milk, and 34 kg/d milk production, 4% of fat content in milk), the in vivo diets were prepared using the FeedExpert software (Rovecom, Hoogeveen, Netherlands). The in vitro experiment was designed in a completely randomized design comprising four diets and three replicates. From d 6 to 10 of each run, fermentation fluid samples were collected under anaerobic conditions from each vessel 3 h before feeding time. The collected fluid samples were analyzed for pH, volatile fatty acids (VFA), ammonia concentration, protozoa, bacteria and methanogen counts. For fatty acids analysis, samples were collected directly from the effluent vessels during bag replacements. Before feeding time (once a day), the fermentation gas was collected in a gas-tight collection bag (Tecobag 81; Tesseraux Container, Bürstadt, Germany) for the methane concentration measurement. The DM degradability was determined by analyzing feed residues in pre-feeding nylon bag samples for the last 5 d of each run (d 6 to 10).

### In vivo experiment using rumen-cannulated dairy cows (Exp. 2)

In Exp. 2, four multiparous cannulated Polish Holstein–Friesian dairy cows (625 ± 20 kg body weight; 4-5^th^ month of lactation) were assigned to two dietary treatments (CON vs. PLS60) with two cows in each treatment in a replicated 2 × 2 crossover design. Based on the results from Exp. 1 (mainly pH, total VFA concentration, methane production, and methanogens population), the CON and PLS diets containing the higher level of PLS, i.e., 60 g/kg DM (PLS60) were implemented in Exp. 2. Cows were fed two times a day. Each period lasted for 36 d, with a 21-d adaptation (where cows were also introduced and accustomed to the conditions of the respiration chambers; temperature 12 to 22 °C and the humidity of 50% to 70%) and a 15-d sampling period (5 d of rumen fluid collection and 10 d for gases collections). The cows were housed in tie stalls with rubber mats with individual feeding and had free access to water and salt blocks during the adaptation and sampling (without the period in which the cows were kept in the respiration chambers). The ruminal fluid was collected from each cannulated cow from three locations (top, bottom, and middle) of the midventral sac of the rumen before morning feeding (0 h) and 3 h and 6 h after morning feeding [10]. Rumen samples (about 400 g/animal) were filtered through a two-layer cheesecloth and analyzed for pH value, ammonia and VFA concentrations, and FA profile. For protozoa counting, about 100 g/animal of the rumen content was mixed with an equal amount of 8% formaldehyde solution (w/w), strained through a two-layer cheesecloth into 10 mL polypropylene tube with a screw cup, and stored at 8 °C in a refrigerator until analysis. Quantification of total bacteria and methanogens was carried out only on rumen fluid sampled 3 h after morning feeding. For microbial analysis, rumen content (300 g) was strained through a two-layer cheesecloth into 100 mL polypropylene box, mixed, transferred into two cryotubes of 4.5 mL and frozen in liquid nitrogen. Samples were stored at − 80 °C until further analyses.

Feed intake, feed residue, and amount of feces were recorded daily from individual cows kept in respiratory chamber (SPA System, Wroclaw, Poland) during the sampling period (d 27 to 36) to determine the total-tract degradability coefficients. The feed and feces subsamples (about 5% wt/wt) were stored at -20 °C for DM, organic matter (OM), neutral detergent fiber (NDF), and CP analyses. The total tract nutrient degradability was calculated as [(nutrient intake – nutrient in feces)/nutrient intake] × 100.

### In vivo experiment using commercial dairy cows (Exp. 3)

In Exp. 3, 16 multiparous lactating Polish Holstein–Friesian dairy cows [600 ± 30.4 kg body weight, 2.4 ± 0.45 parity, 160 ± 32 d in milk, and 33 ± 2.1 kg/d milk production; (mean ± SD)] were assigned to two dietary treatments (CON vs. PLS60) with eight cows in each treatment in a replicated 2 × 2 crossover design. Each period consisted of a 21-d adaptation period followed by a 5-d sampling period with a total of 26 d. Cows were randomly assigned to one of the two dietary groups (*n* = 8): control (CON) and experimental diet (PLS 60) and kept separately in a dedicated area of a barn. The two groups (8 CON and 8 PLS60) had separated access (gate with access control) to a computer-controlled feeder station (De Laval, type FP 204, Tumba, Sweden). The concentrate supplement, contained (as g/kg of DM in concentrate) OM (910), aNDFom (240), CP (17.5), and EE (31), was supplied in both groups at 2.75 kg/d/cow (5 times a day). The rest of the diet was offered twice a day (at 06:00 and 18:00 h) as a partial mixed ration (PMR) in individual feeding boxes located on the feeding table. Due to the limitations of running the experiment under production conditions, the cows were supervised by two workers for 18 h, who ensured that each cow consumed feed from an appropriate feeding box (CON or PLS60). For the remaining 6 h, the feeding boxes were closed. The control PMR contained (g/kg of DM): corn silage (441); alfalfa silage (93), meadow grass silage (103); beet pulp (118), brewer’s grain (108); rapeseed meal (122); mineral and vitamin premix (16) whereas experimental PMR contained (g/kg of DM): corn silage (438); alfalfa silage (29), paulownia leaves silage (68), meadow grass silage (102); beet pulp (117), brewer’s grain (108); rapeseed meal (122); mineral and vitamin premix (16). The nutritive value of the two diets (CON and PLS60) was the same. Cows had access to clean water ad libitum. The dry matter intake was measured daily for the last 5 d (d 22 to 26) of the experiment by weighing the individual amounts of feeds offered and leftovers in the feeding boxes. Feces were individually collected from each cow directly after defecation and the floor was kept clean. Subsamples (about 5% wt/wt) were collected and stored at -20 °C until analysis. Total tract nutrient digestibility was calculated using the method described for Exp. 2. Cows were milked twice a day at 5:30 and 17:30 h in a herringbone milking parlor with 8 milking units, spending a maximum of 1 h/d outside the pen for milking. Milk samples were collected from all cows at each milking during the sampling period (d 22 to 26) based on the proportion of morning and evening yield. The morning milk samples were stored at 4 °C until the evening samples were taken. Subsequently, milk samples were prepared in three equal parts: first part was used to analyze milk basic constituents, second part was stored at -20 °C for FA analysis, and the third part was quickly stored in liquid nitrogen for gene expression analysis.

### Sample analysis

Feed and feces samples were analyzed according to AOAC methods [11] for DM (method no. 934.01), ash (method no. 942.05), CP (Kjel-Foss Automatic 16,210 analyzer, Foss Electric, Hillerød, Denmark; method no. 976.05), ether extract (EE; Soxhlet System HT analyzer; Foss Electric, Hillerød, Denmark; method no. 973.18) and neutral detergent fiber (aNDF) with amylase and sodium sulfite and expressed without residual ash (Fibertech 1020 Analyzer; Foss., Analytical AB, Höganäs, Sweden; method according Van Soest et al. [12]. Organic matter was obtained by subtracting ash from DM. Concentration of total phenolic compounds in the diets (Table 1) was calculated by utilizing data for ensiled paulownia leaves published by Huang et al. [4]. The same batch of ensiled paulownia leaves was used in the present study. The silage pH was determined using a pH meter (Elmetron, Type CP-104, Zabrze, Poland). Ammonia was estimated according to the Nessler method [13]. Lactic, acetic, propionic, and butyric acids were determined using a High Performance Liquid Chromatography (Waters 2690, Santa Clara, CA, USA) equipped with Waters 2487 Dual *λ* detector and Aminex HPX-87H column (300 mm × 7.8 mm, Bio-Rad, Warsaw, Poland). The quantitative and qualitative evaluations of individual peaks were made using the external standard method prepared by mixing individual fatty acids purchased from Supelco (Poznan, Poland) and analysed with the Millennium 2001 software (version 2.15, Waters Corporation, Manchester, England).

The pH of ruminal fluid was measured immediately after sample collection using a pH meter (Elmetron, Type CP-104, Zabrze, Poland). The ammonia concentration was determined using the colorimetric Nessler method and the VFA was analyzed using gas chromatography (GC Varian CP 3380, Sugarland, TX, USA) as described earlier [9, 13]. In the first two experiments, the content of DM degradability (g/kg) was determined by the difference between the initial feed substrate weight and residue weight after incubation.

Protozoa counts in the fermented fluid were carried out under a light microscope (Primo Star 5, Zeiss, Jena, Germany) using an appropriate volume (10 μL for Ophryoscolecidae and 100 μL for Isotrichidae). In Exp. 2, the protozoan genera and species were identified according to size and shape of cells, skeletal plates (if present), macronucleus, and arrangement of ciliature [14]. The methanogens and total bacteria were quantified by fluorescence in situ hybridization (FISH), following the procedure described previously [15] with some modification. Briefly, 6 mL of sterile paraformaldehyde-phosphate buffered saline (PBS; pH 7.2) was added to 2 mL of ruminal fluid in a stomacher bag. The mixture was homogenized in a stomacher (Interscience, Saint-Nom-la-Breteche, France) for 2 min. Afterwards, 2 mL of the homogenized mixture was transferred to the eppendorf tubes and fixed with 4% (w/v) sterile paraformaldehyde-PBS for 3 h at 48ºC. Mild sonication (two times for 30 s using Hielscher Ultrasonics, Teltow, Germany) was done to avoid the formation of clusters and to optimize homogenization of the samples [16]. The prepared samples were pipetted onto 0.22 μm polycarbonate filters (Frisenette K02BP02500) and vacuumed (Vaccum KNF Vacuport-Neuberg). After vacuuming, the filters were transferred onto cellulose disks for dehydration in ethanol series (50%, 80%, and 90%, 3 min each). For each sample, a series of identical filters were prepared to allow the determination of optimal hybridization. Hybridizations were carried out in 50 μL of hybridization buffer (0.9 mol/L NaCl; 20 mmol/L Tris/HCl, pH 7.2; 0.01% SDS) containing oligonucleotide probes for all methanogens (S-D-Arch-0915-a-A-20) and two order-specific probes (S-O-Mmic-1200-a-A-21 Methanomicrobiales and S-F-Mbac-0310-a-A-22 Methanobacteriales) [17]. After hybridization, the filters were washed with a washing buffer (20 mmol/L Tris/HCl, pH 7.2; 0.01% SDS; 5 mmol/L EDTA) for 20 min at 48 °C. The filters were rinsed gently in distilled water, air-dried, and mounted on object glasses with VectaShield (Vector laboratories nr. H-1000) anti-fading agent containing DAPI (4ʹ,6-diamidino-2-phenylindole). To distinguish the total count of bacteria (DAPI) from methanogens in the ruminal fluid, filters were maintained at 4 °C for one h in the dark until visualization using an Axio Imager M2 microscope (Carl Zeiss Iberia, Madrid, Spain). Besides bacteria counts, relative changes in the population of seven selected species (*Ruminococcus flavefaciens, Fibrobacter succinogenes, Streptococcus bovis, Butyrivibrio proteoclasticus, Ruminococcus albus, Butyrivibrio fibrisolvens, Megasphaera elsdenii)* and two genera (*Prevotella* spp., *Lactobacillus* spp*.*) of ruminal bacteria were determined by quantitative real-time PCR (qPCR). For this purpose, total DNA was extracted from the ruminal fluid using QIAamp DNA Stool mini kit (Qiagen GmbH, Hilden, Germany) according to Szczechowiak et al. [9]. Sequences of primers specific to bacterial genera and species are given in a Supplementary data (Table S1). The specificity of primers (Table S1) was confirmed in the GenBank Database using the BLAST program. The quantitative analysis of particular bacteria was performed with a known starting concentration of bacterial DNA (25 ng/μL) using the QuantStudio 12 Flex PCR system (Life Technologies, Thermo Fisher Scientific, Waltham, MA, USA) [9]. The Power SYBR Green PCR Master Mix (Thermo Fisher Scientific, Waltham, MA, USA) was used for PCR amplification. The reaction mixture (final volume of 10 µL) contained 4 µL of the 2 × Mastermix, 25 ng of template DNA and 0.5 mol/L of each primer. Amplification involved one cycle at 95 °C for 10 min for initial denaturation, 45 cycles of 95 °C for 15 s followed by annealing at temperatures depending upon the individual bacteria, and then primer extension at 60 °C for 62 s. The fluorescent product was monitored in the last step of each cycle. To determine the amplicon specificity, melting analysis was performed after a single amplification (0.1 °C × s^−1^ increment from 65 °C to 95 °C with fluorescence collection at 0.1 °C intervals). Additionally, the size of amplicons was verified by gel electrophoresis. The size of total bacterial populations was referred to the calculated copy number concentrations of the 16S rRNA (*rrs*) gene [18]. The absolute abundance of bacterial DNA was expressed as a number of *rrs* gene copies/mL of ruminal sample.

In the first experiment, the methane concentration was determined using gas chromatograph (SRI PeakSimple 310; Alltech, PA, USA) fitted with Carboxen 1000 column (Supelco, Bellefonte, USA) and thermal conductivity detector according to Szumacher-Strabel et al. [19]. In the in vivo experiments, the methane and carbon dioxide concentration were measured using two separate NDIR (nondispersive infrared spectroscopy) systems (one system per gas) operating in the near-infrared spectrum (detector 1210 Gfx Servomex 4100, Servomex, Crowborough, UK). In Exp. 2, two respiration chambers were used for monitoring of methane production. Briefly, two open-circuit respiration chambers (W × L × H: 300 cm × 400 cm × 220 cm; SPA System, Ltd., Wroclaw, Poland) were used to measure CH_4_ and CO_2_ over 10 d (d 27 to 36). During the sampling period (from d-27 to 36 of the experiment), individual cows were transferred into a respiratory chamber by daily rotation in order to determine the direct CH_4_ emission for 23.5 h consecutively. The time of milking (approximately 30 min) that accompanied the morning and evening feedings was not included in the gas emission calculations. Finally, each cow was monitored for 5 d. During the adaptation period (from d 11 to d 20) to decrease stress, cows were getting accustomed to the respiratory chambers. Cows were restrained within the chambers by a neck yoke on a dedicated platform (180 cm × 126 cm) covered with a rubber mat and had free access to fresh water and salt blocks. Emission of CH_4_ and CO_2_ was determined using two NDIR analyzers operating in the near-infrared spectrum (SERVOMEX 4100, SERVOMEX Ltd, UK, detector 1210 Gfx). Measurements were taken every 2-s interval. Two measuring channels were used: the concentration of CO_2_ in the range of 0–2.5% (0–48,450 mg/m^3^) and the CH_4_ concentration in the range of 0–1000 ppm (0–706 mg/m^3^). Samples were collected and ducted to the analyzer via a polyethylene tube with an 8 mm diameter. The sampling rate was 0.6 L/min. Before starting the experiment, analyzers were calibrated using calibration gasses: nitrogen N 5.0 (99,999 vol % purity) and 1210 ppm CH_4_ in nitrogen. The analyzer was equipped with 0.17 L cuvette with 540 mm optical track length for CH_4_ and 0.012 L cuvette with 154 mm optical track length for CO_2_. In Exp. 3, methane and carbon dioxide concentrations were measured by the same type of detectors during the feeding of concentrate in the feeder station for the last 4 d of the experiment [20]. Air samples were continuously collected from the feed bins in the feeder station at 15 L/min via an 8-mm diameter polyethylene tube and connected to detectors.

The FAs concentrations in collected samples (feed, ruminal fluid, and milk) were determined by gas chromatograph (456-GC, Bruker, USA) with fused-silica capillary column (100 m × 0.25 mm; overlaid with 0.25 µm Agilent HP; Chrompack CP7420; Agilent Technologies, Santa Clara, CA, USA) and flame ionization detector [9].

Daily milk yields were recorded using a milk meter (WB Ezi-Test Meter 33 kg; True-Test, Manukau, New Zealand). The milk composition was measured by infrared analysis (MilkoScan 255 A/S N, FossElectric, Hillerød, Denmark). For gene expression in milk somatic cells, total RNA was isolated from 10 mL milk samples frozen in liquid nitrogen using previously published procedure [9]. Briefly, milk samples after thawing at 4 °C were centrifuged in 15 mL tubes for 10 min at 3000 × *g*. The supernatant was discarded, and the pellet was dissolved in 1 mL TriPure reagent (Roche) and incubated 5 min. After incubation, 200 µL of chloroform were added and shaken vigorously for 30 s. After 10 min incubation at room temperature, the sample was centrifuged for 15 min at 12,000 × *g*. The clear phase was transferred to a new tube, 0.5 mL isopropanol was added and incubated for 10 min at room temperature. The next steps involved RNA precipitation with a 75% ethanol and drying on a 40 °C thermoblock. The RNA was resuspended in DEPC treated water following spectrophotometric measurement (Nanodrop c2000, Thermo Scientific, USA) of its concentration and purity. A reverse transcription reaction (RT) was performed using a Transcriptor First Strand cDNA Synthesis Kit (Roche) according to the manufacturer’s protocol. Each sample contained equal concentrations of RNA. The RNA mix composed of RNA (300 ng), random hexameters, oligodT (60 µmol/L and 2.5 mmol/L respectively) and water was incubated at 65 °C for 10 min. Next, reverse transcriptase, RNAse inhibitor, dNTP and buffer were mixed and added to the RNA mix to a final volume of 20 µL. The RT conditions were as follows: 25 °C for 5 min, followed by 42 °C for 45 min and 85 °C for 5 min. Resultant cDNA was stored at − 20 °C until further analyses. The mRNA expressions of six genes encoding enzymes regulating FA metabolism [acetyl-CoA carboxylase 1 (*ACACA*), fatty acid synthase (*FASN*), lipoprotein lipase (*LPL*), stearoyl-CoA desaturase (*SCD*), fatty acid desaturase 1 (*FADS1*) and fatty acid elongase 5 (*ELOVL5*)] were measured in milk somatic cells using previously published primer pairs [21, 22]. Each gene was analyzed in technical duplicates using a LightCycler 480 instrument (Roche Diagnostics, Basel, Switzerland) and a LightCycler 480 Sybr Green I Master reagent (Roche Diagnostics, Basel, Switzerland).

### Statistical analysis

The data of the Exp. 1 (Rusitec) were analyzed using a mixed model procedure (PROC MIXED) of SAS (university edition, version 9.4; SAS Institute, Cary, NC, USA). The dietary treatment was considered as the fixed effect, experimental run as the random effect, and the day (6 to 10 d) as the repeated factor. The linear, quadratic and cubic contrasts were used to determine the effect of PLS dose. In Exp. 2 (cannulated cows), ruminal fermentation and FA data were analyzed using PROC MIXED (ver. 9.4, SAS Institute Inc., Cary, NC) for a crossover design with a model containing group (dietary treatment sequence), period, and treatment as main effects, sampling time as repeated measures, and cow as a random effect. The model for the bacteria, methanogens and degradability analyses contained cow, group, period, and treatment as a main effect. In Exp. 3 (productive dairy cows), the data were subjected to analysis of variance, considering the crossover design, testing the effect of treatment (CON and PLS60), group (dietary treatment sequence), period as fixed effects and cows within a group as a random effect. The analysis of milk production, component yields, and composition was performed on the mean values of the milk variables obtained from two sampling points per day (morning and evening milking). The analysis of FA proportion in the milk as well as expression of six genes was conducted on the mean values of pooled samples from morning and evening milking. The results were tested with an independent *t*-test where the means of both groups were compared through PROC TTEST procedure. The results were considered significant when the *P*-values were lesser than 0.05. All values are shown as the means with pooled standard errors of means.

## Results

### Exp. 1 (Rusitec study)

Ruminal pH increased linearly and quadratically (*P* < 0.01) with increasing concentrations of PLS with the greatest pH at the highest PLS inclusion (Table 2). The inclusion of PLS in diets increased the ammonia concentration linearly and cubically (*P* < 0.01) and total VFA concentration linearly (*P* < 0.01). Molar proportion of acetate and acetate to propionate ratio decreased (*P* < 0.01) linearly; however molar proportions of other VFA (except isovalerate) increased linearly with increasing levels of PLS (*P* < 0.01). Degradability of nutrients was not affected by the PLS inclusion in diets. The total gas production increased (*P* < 0.01) linearly when PLS was included into the diet. Daily methane concentration decreased (*P* < 0.05) linearly, quadratically, and cubically with increasing doses of PLS in diets. Methane concentration per unit of degraded DM decreased linearly and quadratically (*P* < 0.05) as the PLS dose increased. The inclusion of PLS into the diet resulted in changes (*P* < 0.05) of most microbial populations in a linear pattern (Table 3). Total protozoa and Ophryoscolecidae populations decreased linearly (*P* < 0.01) whereas Isotrichidae counts increased linearly (*P* < 0.05) with increasing PLS levels. Total archaea, Methanobacteriales and Methanomicrobiales populations were decreased (*P* < 0.01) linearly by the higher replacement of alfalfa silage with PLS in the diet. The increasing PLS supplementation caused linear increase of *Ruminococcus flavefaciens, Fibrobacter succinogenes, Streptococcus bovis*, *Prevotella* spp., *Butyrivibrio fibrisolvens,* and *Megasphaera elsdenii* (*P* ≤ 0.05) abundances. Besides, *M. elsdenii* showed quadratic and cubical responses (*P* < 0.05).

**Table 2:** The effect of paulownia leaves silage (PLS) on in vitro ruminal fermentation and methane production (*n* = 4) (Exp.1)

**Parameters**^a^	**CON**	**PLS, g/kg DM**^b^			**SEM**	**Contrast**^c^		
		**20**	**40**	**60**		**L**	**Q**	**C**
Rumen fermentation								
Redox potential, mV	-313	-314	-316	-319	1.27	0.22	0.77	0.87
pH	6.28	6.52	6.62	6.71	0.02	< 0.01	< 0.01	0.06
NH_3,_ mmol/L	9.09	9.30	12.5	12.8	0.25	< 0.01	0.89	< 0.01
Total VFA, mmol/L	63.5	64.9	66.5	69.5	0.48	< 0.01	0.19	0.68
VFA, mol/100 mol								
Acetate (A)	60.5	57.8	57.4	55.6	0.37	< 0.01	0.38	0.10
Propionate (P)	16.9	18.3	18.8	19.7	0.21	< 0.01	0.27	0.25
Isobutyrate	0.85	0.96	1.04	1.11	0.02	< 0.01	0.08	0.63
Butyrate	13.1	14.0	14.3	14.6	0.12	< 0.01	0.22	0.44
Isovalerate	2.49	2.44	2.41	2.40	0.04	0.29	0.69	0.96
Valerate	6.05	6.44	6.44	6.59	0.08	< 0.01	0.28	0.29
A/P ratio	3.56	3.19	3.04	2.84	0.05	< 0.01	0.18	0.31
Degradability, g/kg DM								
DM	538	540	562	564	6.07	0.18	0.99	0.52
OM	570	573	593	595	5.70	0.18	0.99	0.52
CP	569	574	578	582	2.74	0.13	0.97	0.93
NDF	437	420	443	440	6.67	0.71	0.64	0.31
Total gas and methane production								
TGP, mL/d	3722	3881	3958	4151	25.7	< 0.01	0.71	0.31
CH_4,_ mmol/L	9.45	8.10	6.08	5.94	0.21	< 0.01	0.03	0.04
CH_4,_ mmol/g DMD	1.58	1.29	0.94	0.92	0.04	0.02	0.03	0.17
CH_4,_ mmol/g NDFD	3.57	3.05	2.26	2.04	0.10	0.26	0.39	0.15

^a^*VFA* volatile fatty acid, *DM* dry matter, *Om,* organic matter, *CP* crude protein, *NDF* neutral detergent fiber, *TGP* total gas production, *DMD* dry matter degradability *NDFD* neutral detergent fiber degradability

^b^*CON* control diet, *PLS* paulownia leaves silage diet, *PLS* was used at 20, 40, and 60 g/kg DM of diet replacing alfalfa silage

^c^*L* linear response, *Q* quadratic response, *C* cubic response. The results are considered to be significantly different at *P* ≤ 0.05

**Table 3:** The effect of paulownia leaves silage (PLS) on in vitro ruminal microbial population (*n* = 4) (Exp.1)

**Parameters**	**CON**	**PLS, g/kg DM**^a^			**SEM**	**Contrast**^b^		
		**20**	**40**	**60**		**L**	**Q**	**C**
Total bacteria, × 10^8^/mL	1.72	1.58	1.63	1.53	0.08	0.20	0.85	0.40
Total protozoa, × 10^3^/mL	14.0	12.3	11.9	10.7	0.25	< 0.01	0.62	0.27
Ophryoscolecidae*,* × 10^3^/mL	13.2	11.5	10.8	9.17	0.27	< 0.01	0.92	0.36
Isotrichidae*,* × 10^3^/mL	0.76	0.79	1.15	1.48	0.05	< 0.01	0.05	0.27
Total archaea, × 10^6^/ mL	3.29	2.86	2.55	2.17	0.13	< 0.01	0.87	0.84
Methanobacteriales, × 10^6^/mL	2.46	2.15	1.96	1.68	0.09	< 0.01	0.93	0.79
Methanomicrobiales, × 10^5^/mL	2.40	2.13	2.02	1.66	0.09	< 0.01	0.79	0.55
Ruminococcus flavefaciens*	0.73	0.65	3.03	5.03	0.80	0.02	0.41	0.75
Fibrobacter succinogenes*	0.19	0.55	0.51	1.83	0.22	0.05	0.33	0.48
Streptococcus bovis*	1.06	2.00	5.61	18.85	2.55	< 0.01	0.11	0.59
*Prevotella* spp.*	3.26	6.88	13.78	20.75	2.06	< 0.01	0.40	0.28
Butyrivibrio proteoclasticus*	1.68	3.18	4.21	6.70	1.06	0.11	0.14	0.53
Ruminococcus albus*	0.21	0.27	1.11	1.42	0.16	0.65	0.13	0.89
Butyrivibrio fibrisolvens*	0.40	0.44	1.52	4.25	1.04	< 0.01	0.98	0.52
*Lactobacillus* spp.*	2.15	3.44	8.24	10.06	0.94	0.20	0.61	0.96
Megasphaera elsdenii*	0.73	0.65	3.03	5.03	0.80	< 0.01	< 0.01	0.02

^a^*CON* Control diet, *PLS* paulownia leaves silage diet

^b^*L* linear response, *C* cubic response

^*^Abundance (log_10_ no. of copies of rrs gene/mL of buffered rumen sample)

### In vivo experiment (using cannulated cows)

Inclusion of PLS resulted in an increase (*P* < 0.01) in pH and ammonia concentration in the rumen. Besides, pH values were post-feeding time dependent (*P* < 0.01) whereas ammonia concentration showed treatment × time interaction (Table 4). The inclusion of PLS decreased the molar proportion of acetate (*P* < 0.01), but increased the molar proportion of propionate (3 h and 6 h after feeding; *P* < 0.01), isovalerate (*P* < 0.05) and valerate (*P* < 0.01). Molar proportions of most individual VFA showed time-dependent variations (*P* < 0.05). A/P ratio was lower in the PLS group compared to the control group in 3 h and 6 h after morning feeding (*P* < 0.01). Ammonia concentration was affected (*P* < 0.01) by time × treatment interaction, which increased in the PLS diet compared with the control diet (*P* < 0.01) at 3 and 6 h, but not at 0 h.

**Table 4:** The effect on replacing alfalfa silage with paulownia leaves silage (60 g/kg) on ruminal fermentation characteristics measured in rumen-cannulated cows (*n* = 4) (Exp. 2)

Variables^1^	0 h^2^		3 h^2^		6 h^2^		Treatment		SEM	*P*-value^4^		
	CON^3^	PLS^3^	CON	PLS	CON	PLS	CON	PLS		T	H	T × H
pH	5.93	6.07	5.92	6.13	6.09	6.18	5.98	6.13	0.02	< 0.01	< 0.01	0.22
NH_3_, mmol/L	8.73^b^	8.57^b^	7.84^b^	11.2^a^	6.75^c^	12.4^a^	7.80	10.7	0.41	< 0.01	0.50	< 0.01
Total VFA, mmol/L	106	108	106	104	107	107	106	106	0.53	0.64	0.33	0.44
VFA, mol/100 mol												
Acetate (A)	64.5^a^	64.4^a^	63.0^a^	60.2^b^	63.1^a^	59.2^b^	63.5	61.2	0.25	< 0.01	< 0.01	< 0.01
Propionate (P)	22.1^c^	21.1^c^	22.4^c^	24.8^a^	23.1^bc^	24.8^a^	22.6	23.6	0.19	< 0.01	< 0.01	< 0.01
Isobutyrate	0.67	0.75	0.70	0.74	0.65	0.77	0.67	0.75	0.03	0.22	0.96	0.85
Butyrate	9.88^c^	10.6^b^	11.1^a^	10.7^b^	10.6^b^	11.8^a^	10.5	11.0	0.12	0.02	< 0.01	< 0.01
Isovalerate	1.10^b^	1.52^a^	1.13^b^	1.80^a^	1.10^b^	1.84^a^	1.11	1.72	0.04	< 0.01	0.03	0.04
Valerate	1.66^b^	1.67^b^	1.66^b^	1.81^a^	1.38^c^	1.86^a^	1.57	1.78	0.03	< 0.01	0.08	< 0.01
A/P ratio	2.93^a^	3.06^a^	2.83^a^	2.47^b^	2.75^a^	2.40^b^	2.84	2.64	0.03	< 0.01	< 0.01	< 0.01
Microbial populations												
Total protozoa, × 10^5^/mL	12.1	10.6	13.3	11.3	14.6	11.9	13.2	11.3	0.17	< 0.01	< 0.01	0.16
Isotrichidae, × 10^3^/mL	5.64^c^	6.27^c^	6.14^c^	16.8^b^	6.38^c^	21.7^a^	6.05	15.1	0.57	< 0.01	< 0.01	< 0.01
Ophryoscolecidae, × 10^5^/mL	12.0	10.5	13.2	11.1	14.5	11.7	13.2	11.1	0.17	< 0.01	< 0.01	0.11
* Dasytricha ruminantium,* × 10^3^/mL	5.10^c^	5.29^c^	5.21^c^	15.5^b^	5.24^c^	19.8^a^	5.18	13.6	0.53	< 0.01	< 0.01	< 0.01
* Isotricha prostoma,* × 10^3^/mL	0.25^d^	0.19^d^	0.49^c^	0.93^b^	0.66^c^	1.37^a^	0.47	0.87	0.05	< 0.01	< 0.01	< 0.01
*Isotricha intestinalis,* × 10^3^/mL	0.24	0.33	0.30	0.43	0.47	0.52	0.34	0.43	0.02	< 0.01	< 0.01	0.48
* Entodinium* spp., × 10^5^/mL	11.9	10.4	13.0	11.1	14.3	11.6	13.1	11.0	0.17	< 0.01	< 0.01	0.12
* Ostracodinium gracil*e, × 10^3^/mL	7.16^c^	5.79^c^	9.69^b^	6.64^c^	12.8^a^	9.60^b^	10.0	7.20	0.24	< 0.01	< 0.01	< 0.01
* Polyplastron multivesiculatum,* 10^3^/mL	1.15^b^	0.70^c^	2.24^a^	0.91^b^	2.45^a^	1.23^b^	1.93	0.94	0.07	< 0.01	< 0.01	< 0.01

^1^*NH*_*3*_ ammonia, *VFA* volatile fatty acid

^2^The ruminal fluid was obtained from each cannulated cow from three locations in the midventral sac of the rumen before morning feeding (0 h), 3 h after morning feeding, and 6 h after morning feeding

^3^*CON* control diet, *PLS* paulownia leaves silage diet

^4^* T* treatment, *H* hours

^a,b,c,d,e^Means with different superscript letters differ significantly (*P* < 0.05) among the treatments and hours in a row

Total Isotrichidae, *Dasytricha ruminantium*, *Isotricha prostoma* and *Isotricha intestinalis* populations increased by feeding the PLS diet. The abundances were affected by treatment × time interaction (*P* < 0.01), except for *Isotricha intestinalis*. *Ostracodinium gracile* and *Polyplastron multivesiculatum* (*P* < 0.01) also were affected by treatment × time interaction. The first three protozoa mentioned were increased by PLS at 3 h and 6 h, but were similar at 0 h; whereas *Ostracodinium gracile* population was greater at 3 h and 6 h, and *P. multivesiculatum* was greater at 0, 3, and 6 h in the PLS diet than in the control diet. The inclusion of PLS in the diet decreased total protozoa, Ophryoscolecidae and *Entodinium* spp. counts (*P* < 0.01).

Feeding PLS to cannulated cows increased (*P* < 0.05) the populations of all bacterial species examined in this study, except *Ruminococcus flavefaciens, Ruminococcus albus* and *Lactobacillus* spp. (Table 5). The decreases in abundances of total archaea, Methanobacteriales and Methanomicrobiales were noted in the experimental group (*P* < 0.01). The inclusion of PLS did not affect degradability of DM, OM, and NDF, but a lower crude protein degradability was observed (*P* < 0.01) in PLS diet. The EE degradability increased due to PLS feeding (*P* < 0.05). Lowered (*P* < 0.01) methane production (g/d) and yield (g/kg DM intake) in PLS diet were noted compared to the CON diet; however, PLS diet did not alter CO_2_ production in the rumen-cannulated cows.

**Table 5:** The effect on replacing alfalfa silage with paulownia leaves silage (60 g/kg) on bacteria, methanogens, methane (CH_4_) production and digestibility measured in rumen-cannulated cows (*n* = 4) (Exp. 2)

Item	Treatments^a^		SEM	*P*-value^b^
	CON	PLS		
Microbial populations				
Ruminococcus flavefaciens*	1.18	10.77	2.79	0.09
Fibrobacter succinogenes*	0.13	0.32	0.04	< 0.01
Streptococcus bovis*	1.07	4.61	0.51	< 0.01
* Prevotella* spp.*	3.81	12.91	0.90	< 0.01
Butyrivibrio proteoclasticus*	2.19	9.97	1.93	0.04
Ruminococcus albus*	0.36	0.39	0.45	0.74
Butyrivibrio fibrisolvens*	0.36	2.82	0.51	0.01
* Lactobacillus* spp.*	0.64	0.58	0.04	0.46
Megasphaera elsdenii*	3.98	16.64	1.82	< 0.01
Total bacteria, × 10^9^/mL	7.15	6.86	0.20	0.51
Total archaea, × 10^8^/mL	6.28	5.28	0.19	< 0.01
Methanobacteriales, × 10^8^/mL	4.33	3.44	0.15	< 0.01
Methanomicrobiales, × 10^7^/mL	3.82	3.21	0.13	< 0.01
Dry matter intake	23.2	22.9	0.08	0.07
Total-tract digestibility ^c^, g/kg DM				
DM	631	618	4.42	0.15
OM	660	654	5.34	0.61
NDF	497	514	10.4	0.46
CP	616	584	6.07	< 0.01
EE	696	747	11.1	0.02
CH_4_, g/d	459	410	9.80	< 0.01
CH_4_, g/kg DMI	22.1	19.5	0.29	< 0.01
CO_2,_ g/d	11,403	12,008	203	0.97
CO_2_, g/kg DMI	504	511	7.38	0.74

^a^*CON* control diet, *PLS* paulownia leaves silage diet; the percentage means of how many percentages of alfalfa was replaced with paulowina silage

^b^The results are considered to be significantly different at *P* ≤ 0.05

^c^*DM* dry matter, *OM* organic matter, *CP* crude protein, *EE* ether extract, *NDF* neutral detergent fiber

^*^Abundance (log_10_ number of copies of *rrs* gene/mL of rumen sample)

In the Exp. 2 (cannulated cows), the ruminal fluid of PLS cows was characterized by altered proportions of selected FAs. The level of C8:0, C10:0, C12:0, C16:0, C16:1, C17:1 and C18:0 decreased (*P* < 0.05; Table 6) whereas the level of C14:1, C:15:0, C:15:1, and C17:0 increased (*P* < 0.01). Regarding the proportion of C8:0, C12:0, C14:1, C:15:0, C:15:1, C16:0, and C16:1, the treatment × time interaction was also observed (*P* < 0.05). The proportion of C18:1 *trans*-10, C18:1 *trans*-11, C18:2 *cis*-9, *cis*-12, C18:2 *cis*-9, *trans*-11, and C18:2 *trans*-10, *cis*-12 increased in both treatment (*P* < 0.01) and time-dependent (*P* < 0.01) manners in PLS treatments. The PLS diet tended to decrease C18:1 *cis*-9 (*P* < 0.05), but increase C18:3n-6 and C18:3 *cis*-9, *cis*-12, *cis*-15 (*P* < 0.01) in treatment-dependent. Sum of unsaturated fatty acids (UFA), monounsaturated fatty acids (MUFA), PUFA, n-6 FA and sum of n-3 FA increased (*P* < 0.01) in the experimental group, which resulted in lower sum of SFA (*P* < 0.01). Time-dependent variation (*P* < 0.01) was observed in all of the above parameters, in addition to the sums from MUFA. The inclusion of PLS increased the sum of *trans* C18:1, sum of medium-chain FA, n-6/n-3 FA ratio and PUFA/SFA ratio (*P* ≤ 0.05). The treatment × time interaction was noticed for the following FAs: C18:1 *trans*-10, C18:2 *cis*-9, *cis*-12, C18:2 *cis*-9, *trans*-11, and C18:2 *trans*-10, *cis*-12 and sum of other FA, SFA, UFA, PUFA, n-6 FA, *trans* C18:1, n-6/n-3 FA ratio, and PUFA/SFA ratio (*P* < 0.05).

**Table 6:** The effect on replacing alfalfa silage with paulownia leaves silage (60 g/kg) on fatty acid (FA) proportion (g/100 g of FA) in ruminal fluid (*n* = 4) (Exp. 2)

Item	0 h		3 h		6 h		Treatment^1^		SEM^2^	*P*-value^3^		
	CON	PLS	CON	PLS	CON	PLS	CON	PLS		T	H	T × H
C8:0	0.13^a^	0.092^b^	0.11^a^	0.089^b^	0.088^b^	0.11^a^	0.11	0.10	0.006	0.02	0.21	< 0.01
C10:0	0.13	0.10	0.14	0.10	0.13	0.10	0.13	0.10	0.005	< 0.01	0.72	0.98
C12:0	0.44^a^	0.10^b^	0.45^a^	0.10^b^	0.38^a^	0.10^b^	0.42	0.10	0.019	< 0.01	0.02	0.04
C14:0	1.12	1.05	1.10	0.96	0.93	0.95	1.05	0.98	0.03	0.04	< 0.01	0.12
C14:1	1.18^b^	1.92^a^	1.14^b^	1.89^a^	1.16^b^	1.58^a^	1.16	1.80	0.05	< 0.01	< 0.01	< 0.01
C15:0	1.17^b^	1.52^a^	1.10^b^	1.54^a^	1.12^b^	1.44^a^	1.13	1.50	0.02	< 0.01	0.01	0.01
C15:1	0.46^b^	0.77^a^	0.47^b^	0.73^a^	0.49^b^	0.66^a^	0.47	0.73	0.02	< 0.01	0.01	< 0.01
C16:0	20.4^a^	19.0^b^	19.7^b^	19.5^b^	20.6^a^	19.3^b^	20.3	19.2	0.15	< 0.01	0.23	0.02
C16:1	0.48^a^	0.22^b^	0.46^a^	0.22^b^	0.32^a^	0.23^b^	0.42	0.22	0.002	< 0.01	0.01	< 0.01
C17:0	0.56	0.60	0.52	0.62	0.52	0.59	0.54	0.60	0.009	< 0.01	0.08	0.07
C17:1	0.12	0.079	0.10	0.10	0.12	0.087	0.11	0.087	0.005	< 0.01	0.95	0.10
C18:0	47.8	45.9	47.4	46.7	46.9	45.1	47.4	45.9	0.42	< 0.01	0.17	0.55
C18:1 *trans-*10	1.36^b^	2.51^a^	1.23^b^	2.57^a^	1.31^b^	2.78^a^	1.30	2.62	0.07	< 0.01	< 0.01	< 0.01
C18:1 *trans-*11	0.41	0.67	0.46	0.70	0.46	0.74	0.45	0.70	0.02	< 0.01	< 0.01	0.36
C18:1 *cis*-9	5.67	5.78	6.06	5.56	6.64	5.96	6.13	5.76	0.14	0.03	< 0.01	0.21
C18:2 *cis-*9 *cis-*12	5.27^c^	7.38^a^	6.52^b^	6.78^b^	6.74^b^	7.47^a^	6.14	7.19	0.14	< 0.01	< 0.01	< 0.01
C18:2 *cis-*9 *trans-*11	0.16^b^	0.86^a^	0.24^b^	0.87^a^	0.21^b^	0.87^a^	0.20	0.87	0.03	< 0.01	< 0.01	0.03
C18:2 *trans-10 cis-*12	0.12^b^	0.16^a^	0.15^b^	0.14	0.15^b^	0.17^a^	0.14	0.16	0.005	< 0.01	< 0.01	< 0.01
C18:3 n-6	0.16	0.17	0.16	0.17	0.16	0.19	0.16	0.18	0.005	< 0.01	0.21	0.61
C18:3 *cis*-9 *cis*-12 *cis*-15	1.29	1.38	1.15	1.34	1.23	1.34	1.23	1.35	0.02	< 0.01	0.01	0.27
Sum of other FA^4^	9.07^c^	9.83^a^	9.80^a^	9.42^b^	9.48^b^	9.90^a^	9.44	9.71	0.12	0.08	0.36	< 0.01
Sum of SFA	74.5^a^	69.4^b^	73.0^a^	70.5^ab^	71.9^a^	68.7^b^	73.2	69.5	0.36	< 0.01	< 0.01	0.02
Sum of UFA	25.5^c^	30.6^a^	27.0^b^	29.5^ab^	28.1^b^	31.3^a^	26.8	30.5	0.36	< 0.01	< 0.01	0.02
Sum of MUFA	17.3	20.9	17.3	20.4	17.9	20.9	17.5	20.7	0.28	< 0.01	0.27	0.65
Sum of PUFA	8.30^b^	9.73^a^	9.47^a^	8.98^b^	9.56^a^	10.1^a^	9.08	9.59	0.15	< 0.01	< 0.01	< 0.01
Sum of n-6 FA	6.47^c^	8.43^a^	8.13^ab^	7.85^b^	8.33^a^	8.98^a^	7.60	8.41	0.17	< 0.01	< 0.01	< 0.01
Sum of n-3 FA	1.29	1.40	1.15	1.32	1.23	1.34	1.23	1.35	0.02	< 0.01	< 0.01	0.58
Sum of *trans* C18:1	2.38^b^	4.99^a^	2.33^b^	5.03^a^	2.37^b^	5.34^a^	2.36	5.11	0.14	< 0.01	< 0.01	< 0.01
Sum of medium-chain FA	26.2	26.9	25.3	26.8	25.9	26.3	25.9	26.7	0.20	< 0.01	0.23	0.09
Sum of long-chain FA	73.6	73.0	74.0	73.0	73.8	73.5	73.8	73.1	0.21	0.01	0.46	0.45
n-6/n-3 FA ratio	5.13^e^	5.57^de^	7.80^a^	6.05^c^	6.65^b^	6.69^b^	6.44	6.09	0.19	0.05	< 0.01	< 0.01
PUFA/SFA ratio	0.11^c^	0.14^a^	0.13^b^	0.13^b^	0.13^b^	0.15^a^	0.13	0.14	0.003	< 0.01	< 0.01	< 0.01

^1^*CON* Control diet, *PLS* paulownia leaves silage diet

^2^*SEM* standard error of means for the main effect

^3^*T* treatment, *H* hour

^4^Other FA include C14:1 *iso,* C14:1 *anteiso,* C15:1 *anteiso,* C16:1 *anteiso*, C17:1 *anteiso,* C18:1 *trans-*6–8, C18:1 *trans-*9, C18:1 *cis-*11, C18:1 *cis-*12, C18:1 *cis-*13, C18:1 *cis-*14, C18:2 *trans*-11 *cis-*15, C20:0, C20:1 *trans,* C21:0, C18:3 *cis*-9 *trans* 11 *cis*-15, C22:0, C23:0, C24:0, and C24:1

^a-e^Means with different superscript letters differ significantly (*P* < 0.05) among the treatments and hours in a row

### In vivo experiment (using commercial dairy cows)

The inclusion of PLS in the diet of dairy cows affected milk composition and ruminal methane concentration (Table 7). The PLS diets decreased protein and lactose yield (*P* < 0.05), however, it did not affect milk yield, energy corrected milk (ECM) and fat yield. The fat content was not affected by replacing alfalfa silage with PLS, but PLS diet decreased protein and lactose content (*P* < 0.05). Milk urea concentration was increased (*P* < 0.05) by PLS feeding. Methane concentration in the exhaled gas decreased (*P* < 0.01) about 14% in the PLS group compared to the CON group.

**Table 7:** The effect on replacing alfalfa silage with paulownia leaves silage (60 g/kg) on milk production performance and methane concentration of commercial dairy cows (*n* = 16) (Exp. 3)

Item	Treatments^a^		SEM	*P*-value^b^
	CON	PLS		
DM intake	23.5	22.9	0.16	0.11
Milk yield				
Milk, kg/d	33.9	32.5	0.46	0.08
ECM^c^, kg/d	35.5	33.8	0.67	0.17
Fat, g/d	1349	1201	68.2	0.23
Protein, g/d	896	831	16.5	0.02
Lactose, g/d	1296	1189	23.5	0.01
Milk composition				
Fat, g/kg	43.8	44.0	0.99	0.91
Protein, g/kg	34.1	33.4	0.16	0.03
Lactose, g/kg	49.3	47.9	0.28	0.01
Urea, mg/L	224	249	3.06	0.02
Methane, µg/L	211	185	7.1	< 0.01

^a^*CON* Control diet, *PLS* Paulownia leaves silage diet

^b^The results are considered to be significantly different at *P* ≤ 0.05

^c^Energy corrected milk calculated according the following equation: ECM = milk yield (kg) × (38.3 × fat (g/kg) + 24.2 × protein (g/kg) + 783.2)/3.140 [23]

**Fig. 1:**
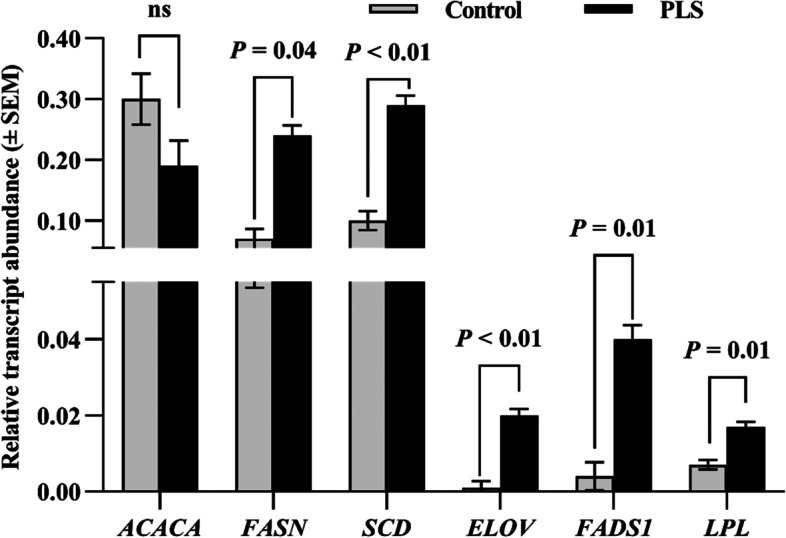
The effect of replacing diet with paulownia leaves silage (PLS: 60 g/kg) on expression of acetyl-CoA carboxylase 1 (*ACACA*), fatty acid synthase (*FASN*), stearoyl-CoA desaturase (*SCD*), fatty acid elongase (*ELOVL*), fatty acid desaturase 1 (*FADS1*), and lipoprotein lipase (*LPL*) genes in the milk of lactating cows (ns—not significant). (Exp. 3)

The PLS diet increased proportions of C15:0, C16:1, C18:2 *cis*-9 *trans*-11, C18:3 *cis*-9 *cis*-12 *cis*-15 and C20:4 n-6 (*P* < 0.01) (Table 8). The proportions of C18:1 *trans*-10, C18:1 *trans*-11, and the sum of *trans*-C18:1 was decreased (*P* < 0.05) by PLS feeding to dairy cows. The PLS diet decreased the total SFA (*P* < 0.05) proportion, but increased the total UFA (*P* < 0.05) and PUFA (*P* < 0.05) proportions. The PUFA/SFA ratio was higher (*P* < 0.05) in the PLS group than in the CON group, but the n-6/n-3 FA ratio was lower (*P* < 0.01) in the PLS group than in the CON group. Supplementation of PLS increased desaturase indices of C14:1, C16:1, and rumenic acid/(vaccenic acid + rumenic acid) (*P* < 0.05). Furthermore, the PLS affected the relative transcript abundances of five out of six analyzed genes. Replacing alfalfa silage with PLS resulted in increased mRNA expressions of all genes (*P* < 0.01) except *ACACA* gene (Fig. 1).

**Table 8:** The effect of replacing alfalfa silage with paulownia leaves silage (60 g/kg) on milk fatty acids (FA) composition (g/100 g FA) and desaturation (DI) of milk of dairy cows (*n* = 16) (Exp. 3)

Item^a^	Treatment^b^		SEM	*P*-value^c^
	CON	PLS		
Saturated FA				
C8:0	0.94	0.92	0.01	0.41
C10:0	2.86	2.91	0.08	0.78
C12:0	3.80	3.75	0.07	0.76
C14:0	11.8	11.5	0.13	0.22
C15:0	1.46	1.59	0.02	< 0.01
C16:0	34.3	33.1	0.44	0.16
C18:0	11.5	11.6	0.13	0.65
Monounsaturated FA				
C16:1	1.33	1.74	0.05	< 0.01
C18:1 *trans*-10	0.53	0.44	0.01	< 0.01
C18:1 *trans*-11	0.60	0.51	0.02	0.02
C18:1 *cis*-9	21.3	22.6	0.32	0.06
Polyunsaturated FA				
C18:2 *cis*-9 *cis*-12	3.23	3.16	0.03	0.38
C18:2 *cis*-9 *trans*-11	0.56	0.72	0.01	< 0.01
C18:2 *trans*-10 *cis*-12	0.11	0.12	0.003	0.13
C18:3 *cis*-9 *cis*-12 *cis*-15	0.39	0.47	0.01	< 0.01
C20:4 n-6	0.13	0.16	0.002	< 0.01
C20:5 n-3	0.058	0.062	0.004	0.30
C22:6 n-3	0.051	0.057	0.002	0.19
Other FA^c^	4.91	4.73	0.06	0.56
Total FA				
Sum of SFA	67.9	66.2	0.39	0.04
Sum of UFA	32.1	33.8	0.39	0.04
Sum of MUFA	27.4	28.8	0.35	0.05
Sum of PUFA	4.66	4.90	0.05	0.03
Sum of n-6 FA	3.98	3.92	0.05	0.51
Sum of n-3 FA	0.51	0.60	0.01	< 0.01
Sum of *trans*-C18:1	1.69	1.53	0.02	< 0.01
Sum of MCFA	52.9	51.6	0.47	0.16
Sum of LCFA	43.2	44.6	0.47	0.18
PUFA/SFA ratio	0.07	0.08	0.001	0.02
n-6/n-3 FA ratio	7.89	6.76	0.12	< 0.01
DI C14:1/(C14:0 + C14:1)^d^	0.28	0.30	0.004	0.01
DI C16:1/(C16:0 + C16:1)	0.037	0.051	0.002	0.02
DI C18:1/(18:0 + C18:1)	0.65	0.66	0.003	0.06
DI RA/(VA + RA)	0.48	0.59	0.01	< 0.01

^a^*SFA* saturated fatty acids, *UFA* unsaturated fatty acids, *MUFA* monounsaturated fatty acids, *PUFA*, polyunsaturated fatty acids, *MCFA* medium-chain fatty acids, *LCFA* long-chain fatty acids, *RA* rumenic acid (C18:2 *cis*-9, *trans*-11), *VA* vaccenic acid (C18:1 *trans*-11)

^a^*CON* control diet, *PLS* paulownia leaves silage diet

^b^The results are considered to be significantly different at *P* ≤ 0.05

^c^Other FA include C14:1, C15:1. C17:0, C17:1, C18:1 *trans*-6–8, C18:1 *trans*-9, C18:1 *cis*-11, C18:1 *cis*-12, C18:1 *cis*-13, C18:1 *cis*-14, C19:0, C20:0, C20:1 *trans,* C18:3 n-6, C21:0, C20:2, C22:0, C20:3 n-6, C22:1 n-9, C20:3 n-3, C23:0, C22:2, C24:0 and C24:1

^d^DI C14:1, DI C16:1, DI C18:1, DI RA/(VA + RA) were calculated according to Bryszak et al. [20]

## Discussion

The present study aimed at assessing the effect of replacing alfalfa silage with PLS in the dairy cow diet on ruminal fermentation processes such as VFA profile, methanogenesis, and biohydrogenation. We have demonstrated a beneficial effect of new forages PLS on ruminal fermentation processes and milk quality without a negative impact on milk production performance.

### Dynamics of microorganism populations (bacteria, methanogens, protozoa) and ruminal fermentation characteristics (pH, ammonia, VFA, methanogenesis)

The dietary use of paulownia leaves has been studied for pigs, small ruminants, rabbits, and birds [6, 7] but no study has been conducted in high-producing dairy cows. The present study investigated ruminal fermentation, microbiota, milk production performance, and methane production variables. We recently published promising results of PLS on the ruminal environment [4]. The advantage of PLS over the fresh paulownia leaves resulted from high nutritive value (comparable to alfalfa) as well as high content of bioactive phenolic compounds (BAC), including phenolic acid (47 g/kg DM) and flavonoid (13 g/kg DM) contents [4]. The influence of PLS on ruminal fermentation characteristics was likely due to the high content of BAC, such as phenolic acids and flavonoids. The present in vitro and in vivo study is a direct continuation of previous work [4]. The intake of phenolic acid and flavonoid in case of the highest PLS dose (60 g/kg DM) was 65 g/d/cow and 18 g/d/cow, respectively. Dietary BAC can exert modulatory or even antimicrobial effects on ruminal microbial populations and thus the basic ruminal characteristics [24, 25]. Therefore, higher ruminal pH (*P* < 0.01) of PLS diets can be partly explained by BAC activity, which decreased (*P* < 0.01) the acetate-to-propionate ratio due to an increase of the numbers of lactate-consuming and propionate- producing bacteria preventing ruminal pH reduction [26]. In the current study, although population of *Streptococcus bovis* increased in the PLS groups (*P* < 0.01), its growth may not cause lactic acid accumulation in the ruminal fluid and thus pH reduction [27]. Increased pH was probably linked to a higher abundance of *Megasphaera elsdenii* (*P* < 0.01) in the PLS diets as noted both in the present in vitro and in vivo experiments. *M. elsdenii* utilizes lactic acid as the main source of energy and thus can protect other ruminal microorganisms from the negative impact of low pH resulting from lactic acid accumulation [28]. High pH positively affects the adhesion of fibrolytic bacteria to the feed particles and thus their degradation [29]. This phenomenon was confirmed in the present study which demonstrated an increase (*P* < 0.01) in the population of some bacteria species such as *Butyrivibrio fibrisolvens* and *Prevotella* spp. in response to PLS supplementation. Zhan et al. [30] showed that flavonoids (from 20 to 100 mg/kg body weight) increased *B. fibrisolvens* population and feed digestion through changes in the population of ruminal microorganisms. In the present experiment on cannulated cows, flavonoids were supplemented at the rate of 29 mg/kg of body weight, which corresponded to the lower range of Zhan et al. [30] study.

Besides changes in pH, PLS diet increased ammonia concentration (*P* < 0.01), both in vitro and in vivo. This observation can be explained by higher ruminal CP degradation compared to the control with non-synchronization of available energy and ammonia in the ruminal fluid [4, 31]. On the other hand, the CP total-tract degradability was reduced (*P* < 0.01) in the PLS cows. Such difference may result from different degradation of protein or distinct solubility of feed protein from alfalfa and paulownia leaves silages [4]. Changes in the observed ammonia concentration may also be associated with alterations in the number of protozoa and bacteria. Although lower protozoa can decrease ammonia concentration due to reduced recycling of engulfed bacterial protein by protozoa, decreased protozoal number may increase bacterial activity or growth in the rumen. In the current study, Ophryoscolecidae decreased (*P* < 0.01), whereas Isotrichidae protozoa significantly increased (*P* < 0.01) in the PLS diet. A previous study indicated that Ophryoscolecidae protozoa have higher bacterivory activity [32]. Thus, a decrease in the number of Ophryoscolecidae could increase selected bacterial populations. In the current study, numbers of proteolytic bacteria such as *Prevotella* spp*.* (*P* < 0.01) and *B. fibrisolvens* (*P* < 0.05) in PLS diets increased considerably, both in vitro and in vivo. *Prevotella* spp. in the rumen are the predominant proteolytic bacteria with diverse and broad range of peptidase activities and represent 20% to 60% of the bacterial abundance [33]. Therefore, it seems that the PLS diet increased proteolytic activity in the rumen, resulting in greater ammonia concentration in the rumen.

Inhibition of ruminal methanogenesis is usually associated with increase in propionate concentration due to competition for hydrogen. High levels of metabolic hydrogen in the rumen may shift fermentation towards propionate by *Prevotella* spp*.* that increased (*P* < 0.01) in the PLS diets. Ruminal *Prevotella* species use different pathways for propionate production utilizing metabolic hydrogen via succinate or acrylate pathways for fermentation of sugars and lactate [34]. Moreover, Seradj et al. [35] suggested that elevated propionate can result from flavonoid supplementation (0.2 mg/g DM), which reduced methane production (*P* < 0.01) and population of methanogenic archaea (*P* < 0.01), and also increased (*P* < 0.01) population of *M. elsdenii* which has been confirmed in the present study.

In our previous in vitro study, paulownia leaves with high total polyphenols content (31 or 35 g/kg DM) lowered ruminal methane production and methanogen populations without affecting substrate degradability and volatile fatty acid concentrations [8]. Similar results were also noted in the current experiments. Besides, the addition of flavonoid-rich plants may impair the growth of some protozoa-associated methanogens [36]. It has been estimated that protozoa-associated methanogens are responsible for up to 37% of methane production [37]. In the present study, results obtained from rumen-cannulated dairy cows verified those from the in vitro experiment. This included reduced methane emissions (*P* < 0.01), increased pH (*P* < 0.01) and ammonia concentration (*P* < 0.01), elevated propionate and butyrate concentrations (*P* < 0.05), decreased methanogens (*P* < 0.01) and Ophryoscolecidae numbers (*P* < 0.01), and increased Isotrichidae population (*P* < 0.01). Differences in protozoa behavior in the ruminal fluid from rumen-cannulated cows (Exp. 2) may suggest their distinct reaction to the BAC of PLS origin.

Another effect of BAC x microorganisms’ interaction is the increase (*P* < 0.01) in the population of *Fibrobacter succinogenes*. The increase in *F. succinogenes* and *B. fibrisolvens* populations might be due to the presence of phenolic acids in PLS. Some phenolic acids such as hydroxycinnamic acid, syringic acid, and p-hydroxybenzoic acid were found to stimulate specific bacterial populations in a dose-dependent manner [38]. Though high concentrations of polyphenols are toxic to ruminal bacteria, a low concentration of polyphenol extract was also found to stimulate the *F. succinogenes* in the rumen [39]. According to Mitsumori et al. [34], elevated numbers of *F. succinogenes* known as non-H_2_ producers may mitigate methane production through H_2_ restriction. Zhang et al. [40] reported that increased propionate and less acetate negatively affect the abundance of *F. succinogenes* and *B. fibrisolvens*. We did not observe such relations regarding *F. succinogenes* and *B. fibrisolvens* population but we noticed decreased acetate (*P* < 0.01) and increased propionate concentration (*P* < 0.01) in ruminal fluid in in vitro and in vivo studies.

### Dynamics in bacteria populations, rumen (FA) and milk parameters (basic composition, FA, transcript expression)

The present study revealed a tendency of reduction in milk production and a decrease in protein and lactose yield (*P* < 0.05) in response to PLS treatment. Khorsandi et al. [1] also reported decreased milk protein and lactose yield after using 120 g/kg DM of pomegranate by-product silage, rich in phenolic compounds (49 g/kg DM). The inclusion of forages rich in phenolic compounds can decrease CP digestibility, as was observed in the current studies, and therefore may negatively affect CP ruminal and post-ruminal digestion. Moreover, in our previous study, the rapidly soluble fraction of protein degradation was higher in PLS than in alfalfa silage, which indicates an imbalance of available energy deficit accompanying increased ammonia concentration in the ruminal fluid [4]. Higher ammonia levels in the ruminal fluid may interact with milk parameters and result in increased urea content (*P* < 0.05) in milk when the diet was supplemented with PLE [41]. Considering this, it may be important to balance the dairy cow rations for easily fermentable nonstructural carbohydrates such as concentrates.

With regard to the fatty acids, an increase in C15:0 proportion (*P* < 0.01) in the ruminal fluid and milk suggests higher activity of *F. succinogenes.* These microorganisms are responsible for synthesis of some FAs, including odd chain (mainly C15:0) and branched-chain FAs [42]. The PLS diet also modulated biohydrogenation of long chain FA such as C18:2 *cis*-9, *cis*-12. According to Szczechowiak et al. [9], BAC derived from lingonberry (*Vaccinium vitis-idaea*) affected the biohydrogenation process with an increase in CLA isomer (C18:2 *cis-9*, *trans*-11) and vaccenic acid (C18:1 *trans*-11) as intermediates, which was also observed in the current study. The amount of C18:2 *trans*-10, *cis*-12 in the ruminal fluid may be influenced by the activity of *M. elsdenii* as shown by Kim et al. [43]. The amount of this conjugated linoleic acid isomer (CLA), and also α-linolenic acid is usually low in milk [44]. However, we did not observe similar changes in the milk.

In case of two FAs, markers of the biohydrogenation process such as C18:1 *trans*-11 and C18:2 *cis*-9 *trans*-11, parallel changes were noticed in ruminal fluid and in milk. Synthesis of these two isomers is dependent on the activity of *B. fibrisolvens*, one of the dominant group A bacteria in ruminal biohydrogenation responsible for transforming UFA into SFA [39, 45]. Despite an increase in abundance of *B. fibrisolvens* in ruminal fluid of the PLS group, no elevation in SFA in favor of UFA was noticed in the current study. This may be due to the activity of ∆9-desaturase affecting proportion of the C18:1 *trans*-11 [20]. On the other hand, BAC may reduce ∆9-desaturase activity, which may lead to the reduction of the C18:1 *trans*-11 in the milk [46]. Another factor affecting concentration of the C18:1 *trans*-11 is the activity of the *B. proteoclasticus,* a B bacteria group capable of efficiently biohydrogenating PUFA to SFA [47]. *B. proteoclasticus* is recognized as an important bacterium that converts C18:1 *trans*-11 to C18:0 and is one of the most sensitive ruminal bacteria to changes in dietary PUFA [48, 49]. Despite an increase in the population of *B. proteoclasticus* in response to PLS, the proportion of C18:1 *trans*-11 did not reduce. Elevated PUFA content was also not related to the reduction of this bacterial species. Considering the complexity of ruminal processes, some other factors cannot be ruled out.

Milk quality in terms of FA composition also depends on the activity of genes being expressed in the mammary gland. We investigated the transcript expression of six genes controlling FA metabolism. The relative transcript abundance of 5 genes increased in response to PLS. The fatty acid synthase (FASN), a multifunctional protein, is responsible for de novo biosynthesis of long-chain PUFAs [50]. An increase in the *FASN* transcript level was not accompanied by an elevation in long-chain FAs although the total and n-3 PUFA content increased, thus improving milk quality. Another factor affecting the elevation of the mentioned PUFAs may be the higher transcript level of the *ELOVL5* gene. This gene encodes a protein responsible for extending long-chain PUFA [51, 52]. According to those authors, an increase in mRNA level of *ELOVL5* gene elevated both n-6 and n-3 PUFA content in milk. Lack of the *ELOVL5* effect on the n-6 PUFA can be linked to increased transcript abundance of *FADS1,* a gene limiting the n-3/n-6 ​ rate of ​PUFA synthesis [53]. In our study, a major increase was observed for n-3 FAs (e.g., C18:3 *cis-*9, *cis*-12, *cis*-15) whereas the proportion of only C20:4 n-6 increased without changing the total n-6 PUFA. We did not observe a limitation of the C18:2 *trans*-10, *cis*-12 proportion although the increased abundance of the *LPL* mRNA was noticed. Unlike in the present study, Bryszak et al. [54] described the decreased level of the *LPL* transcript accompanied by the reduced proportion of this FA. Milk quality is also influenced by MUFA content, especially C18:1 *cis-*9, which is influenced by the *SCD* gene-regulating de novo synthesis of endogenous FAs [55]. Despite a higher transcript content of *SCD* gene in the PLS group, only an increasing trend of C18:1 *cis-*9 proportion was observed for PLS diet.

## Conclusions

Under the conditions of the present study, PLS (60 g/kg DM) reduced methanogenesis, beneficially modulated ruminal fermentation and biohydrogenation processes without a negative impact on milk production performance of lactating dairy cows. Dietary PLS also improved milk FA profile including greater proportions of total UFA, PUFA, conjugated linoleic acid, and C18:1 *trans-*11 along with reduction of n6/n3 ratio. The only negative impact of PLS was an increased ruminal ammonia concentration affecting milk urea content. For this reason, a better energy and protein synergy in PLS-containing diets would be required.

## Supplementary Information

**Additional file 1:** **Table S1.** The sequences of primers specific to the analyzed bacteria species.

## Data Availability

Data available by the Author by request (adam.cieslak@up.poznan.pl).

## Abbreviations

aNDF: Ash free NDF
A/P ratio: Acetate/Propionate ratio
BAC: Biologically active compounds
BH: Biohydrogenation
CH_4_: Methane emission
CO_2_: Carbon dioxide emission
CLA: Conjugated linoleic acid
CP: Crude protein
D∆: Desaturation ∆ at –*n*
DI: Desaturation index
DM: Dry matter
DMD: Dry matter digestibility
DMI: Dry matter intake
DNA: Deoxyribonucleic acids
EE: Ether extract
EI: Elongase index
ELOVL5: Fatty acid elongase 5
FA: Fatty acids
FADS1: Fatty acid desaturase 1
FASN: Fatty acid synthase
FISH: Fluorescence in situ hybridization
IVDMD: In vitro dry matter digestibility
LA: *α-*Linoleic acids
LCFA: Long chain fatty acids
LT: Longissimus thoracis
LNA: *α-*Linolenic acids
LPL: Lipoprotein lipase
MCFA: Medium chain fatty acids
mRNA: Messenger-RNA
MUFA: Monounsaturated fatty acids
NDFD: Neutral detergent fibre digestibility
NH_3_: Ammonia
OM: Organic matter
PBS: Phosphate-buffered saline; paulownia leaves silage
PLS60: Paulownia leaaves silege; Diets containing 60 g/kg DM of PLS
PUFA: Polyunsaturated fatty acids
qPCR: Quantitative PCR
RA: Rumenic acid
RT: Transcription reaction
Rusitec: Rumen simulation technique
SCD: Stearoyl-CoAdesaturase
SFA: Saturated fatty acids
SPE: Solid phase extraction
TI: Thrombogenicity
UFA: Unsaturated fatty acids
VA: Vaccenic acid
VFA: Volatile fatty acids
