# Supplementary material for: The effect of ensiled paulownia leaves in a high-forage diet on ruminal fermentation, methane production, fatty acid composition, and milk production performance of dairy cows
Source: J Anim Sci Biotechnol. 2022 Aug 12;13:104. doi: 10.1186/s40104-022-00745-9 (PMC9373331; doi:10.1186/s40104-022-00745-9)
Supplement: Supplementary file 1 — Additional file 1: Table S1. The sequences of primers specific to the analyzed bacteria species. [file 40104_2022_745_MOESM1_ESM.docx]

Table S1. The sequences of primers specific to the analyzed bacteria species

| Species | Primer sequences (5’ to 3’) | Reference |
| --- | --- | --- |
|  |  |  |
| *Ruminococcus flavefaciens* | F: CGAACGGAGATAATTTGAGTTTACTTAGG  R: CGGTCTCTGTATGTTATGAGGTATTACC | [1] |
| *Fibrobacter succinogenes* | F: GTTCGGAATTACTGGGCGTAAA  R: CGCCTGCCCCTGAACTATC | [2] |
| *Streptococcus bovis* | F: TTCCTAGAGATAGGAAGTTTCTTCGG  R: ATGATGGCAACTAACAATAGGGGT | [3] |
| *Butyrivibrio proteoclasticus* | F: TCCTAGTGTAGCGGTGAAATG  R: TTAGCGACGGCACTGAATGCCTA | [4] |
| *Ruminococcus albus* | F: CCCTAAAAGCAGTCTTAGTTCG  R: CCTCCTTGCGGTTAGAACA | [5] |
| *Butyrivibrio fibrisolvens* | F: ACACACCGCCCGTCACA  R: TCCTTACGGTTGGGTCACAGA | [6] |
| *Megasphaera elsdenii* | F: AGATGGGGACAACAGCTGGA  R: CGAAAGCTCCGAAGAGCCT | [3] |
| Prevotella spp. | F: GAAGGTCCCCCACATTG  R: CAATCGGAGTTCTTCGTG | [3] |
| Lactobacillus *spp.* | F: TATGGTAATTGTGTGNCAGCMGCCGCGGTAA | [7] |
|  | R: AGTCAGTCAGCCGGACTACHVGGGTWTCTAAT |  |

1. Poeker SA, Geirnaert A, Berchtold L, Greppi A, Krych L, Steinert RE, et al. Understanding the prebiotic potential of different dietary fibers using an in vitro continuous adult fermentation model (PolyFermS). Sci Rep. 2018;8:4318. <https://doi.org/10.1038/s41598-018-22438-y> .
2. Denman SE, McSweeney CS. Development of a real-time PCR assay for monitoring anaerobic fungal and cellulolytic bacterial populations within the rumen. FEMS Microbiol Ecol. 2006;58:572–82. <https://doi.org/10.1111/j.1574-6941.2006.00190.x>.
3. Li M, Penner GB, Hernandez-Sanabria E, Oba M, Guan LL. Effects of sampling location and time, and host animal on assessment of bacterial diversity and fermentation parameters in the bovine rumen. J Appl Microbiol. 2009;107:1924–34. <https://doi.org/10.1111/j.1365-2672.2009.04376.x> .
4. Potu RB, AbuGhazaleh AA, HastingsD, Jones K, Ibrahim SA. The effect of lipid supplements on ruminal bacteria in continuous culture fermenters varies with the fatty acid composition. J Microbiol. 2011;49:216–23. <https://doi.org/10.1007/s12275-011-0365-1>.
5. Wang RF, Cao WW, Cerniglia CE. PCR detection of Ruminococcus spp. in human and animal faecal samples. MolCell Probes. 1997;11:259–65. <https://doi.org/10.1006/mcpr.1997.0111>.
6. Yu Y, Lee C, Kim J, Hwang S. Group-specific primer and probe sets to detect methanogenic communities using quantitative real-time polymerase chain reaction. Biotechnol Bioeng. 2005;89:670–9. <https://doi.org/10.1002/bit.20347>.
7. Zeng J, Bian Y, Xing P, Wu QL. Macrophyte species drive the variation of bacterioplankton community composition in a shallow freshwater lake. Appl Environ Microbiol. 2012;78:177–84. <https://doi.org/10.1128/AEM.05117-11>.
